# Mapping Allergic Diseases in Sub-Saharan Africa

**DOI:** 10.3389/falgy.2022.850291

**Published:** 2022-02-17

**Authors:** Ofilia Mvoundza Ndjindji, Joel Fleury Djoba Siawaya

**Affiliations:** Service Laboratoire, Unité de Recherche et de Diagnostics Spécialisés, Centre Hospitalier Universitaire Mère-Enfant, Fondation Jeanne EBORI (URDS-CHUMEFJE), Libreville, Gabon

**Keywords:** mapping-allergies, allergy-diagnosis, allergens, precise-medicine, Africa

## Abstract

The consensus is that allergic diseases are increasing in Africa. However, this paradigm shift has not yet been translated into practice. Focused on infectious diseases (malaria, tuberculosis, HIV), health policies in Sub-Saharan Africa have often neglected the diagnosis and management of allergies. Allergic disease mapping is crucial to grasp the full extent of Africa's allergic diseases' impact. This mapping will require diverting resources to diagnose and study allergies, even more at the dawn of precision medicine.

## Typology of Allergens Sensitization in Africa

In constant increase globally, allergies and allergic diseases are no longer “a rare fact” in Africa as we thought (1, 2). The International Study of Asthma and Allergies in Childhood Phase III that included 16 African countries (Algeria, Cameroon, Congo, Congo (RDC), Ethiopia, Gabon, Ivory Coast, Kenya, Morocco, Nigeria, Reunion Island, South Africa, and Tunisia) (1) and a systematic review based on data from 11 African countries (including Botswana, Democratic Republic of Congo, Ghana, Kenya, Morocco, Mozambique, Nigeria, South Africa, Tanzania, Tunisia, and Zimbabwe) (2), highlight that allergies are emerging disease in Sub-Saharan Africa. In 1999, a study realized on suspected allergy cases in Côte d'Ivoire showed that 56.4%, 30.7%, 23.5%, 8.5%, 2.8%, and 1.4% were, respectively, sensitized to mites, cockroaches, molds, pets' danders (dog and cat), foods (rice, peanut, and soy) and latex allergens. In addition, 52% of cases were polysensitized (3). More than a decade later, studies done in Ghana and Cameroun confirmed the hierarchy of allergens in the sub-Saharan context, with mites (51%), cockroaches (59%), pets' danders (15%), and foods related allergies (8%) as the main allergens (4, 5). High sensitization of Africans to mites (5–11), and cockroaches (5, 7, 8, 10) has been reported in many studies. Moreover, it is essential to emphasize that most studies on hypersensitivity to allergens have been carried out in the context of asthma.

Food allergies are not as well-documented as mites, cockroaches, or even pets' dander allergies. Nevertheless, existing data suggest that the burden of food allergies in Africa should not be neglected or undermined. Food allergies represent 5% to almost 50% of allergic reactions in Africa (2, 12–14).

## Allergies Diagnosis and Investigation in Patients Suspected of Allergic Diseases in Africa

Existing studies revealed that a limited number of studies on allergies in Africa are based on precise diagnostic testing. Most studies reflect self-reported symptoms or prick-test-based sensitization to food, which raises the question of the reliability and precision of the reported data. For example, a study in Ghana clearly showed the gap between reported adverse reactions to foods and the results of allergy tests. Indeed, 11% of participating children reported adverse reactions to foods, and only 5% showed a positive allergy test (13). Moreover, the skin prick test widely used to diagnose and investigate allergies in Africa has a limit. Its limit resides in the fact that a positive person may have been just sensitized rather than genuinely allergic (14). In addition, most of the time, allergies diagnosis is solely based on medical records and do not involve confirmatory test.

## Precision Medicine Prospect in Africa

Regardless of the disease, every patient may have different clinical features, treatment responses, and disease outcomes due to genetic, epigenetic, and environmental factors. Precision medicine, a patient-tailored disease treatment approach, requires appropriate biomarkers and tools to guide the diagnosis and define disease endotypes (15). A stringent and precise diagnostics is required for the better care of allergic patients (16).

To take up the challenge of precise medicine or simply improve the care of allergic patients, Sub-Saharan Africa should develop capacities to diagnose and investigate allergies with precision. Mapping allergic disorders in the African context and implementing precise diagnostic tools will provide essential information for implementing the first and most simple personalized care of allergic subjects. Indeed, the first advice to patients with allergic disease is to avoid exposure to allergens that induce symptoms of their disease. To give that advice, one should identify with precision the disease-causing allergens. Also, mapping and characterizing allergic disease endotypes in the African context would probably help achieve precision medicine in Africa's allergic diseases. First, however, fundamental questions need to be answered in mapping allergic disease endotypes in sub-Saharan Africans.

1. Is highly melanized skin, which protects black people from the effects of sun UV-rays, influence UV-rays' activation of vitamin D?
2. Is vitamin D deficiency in African populations a critical risk factor for developing allergic diseases?
3. What are the biochemical characteristics (micro-environment) of asymptomatic sensitized people and others that progress to allergy etc.?
4. What is the real influence of parasitic worm infections on allergy and its diagnosis in Sub-Saharan Africa?

## Concluding Remarks

Allergy research and capacity building need to be scale-up in Africa to provide the most effective diagnosis, disease monitoring, and patient care for those affected.

## Author Contributions

OM and JD conceptualized the topic and wrote the manuscript. Both authors approved the submitted manuscript.

## Conflict of Interest

The authors declare that the research was conducted in the absence of any commercial or financial relationships that could be construed as a potential conflict of interest.

## Publisher's Note

All claims expressed in this article are solely those of the authors and do not necessarily represent those of their affiliated organizations, or those of the publisher, the editors and the reviewers. Any product that may be evaluated in this article, or claim that may be made by its manufacturer, is not guaranteed or endorsed by the publisher.
